# Supplementary material for: Using community-based participatory approaches to improve access to mass drug administration for trachoma elimination in a pastoral conflict area of Kenya
Source: PLoS Negl Trop Dis. 2024 Nov 11;18(11):e0012653. doi: 10.1371/journal.pntd.0012653 (PMC11581410; doi:10.1371/journal.pntd.0012653)
Supplement: Attachment — Submitted filename: Response to reviewers_241024.pdf [file pntd.0012653.s003.pdf]

24th October, 2024

Dear Editor,
PLOS Neglected Tropical Diseases

Re- submission (submission (PNTD-D-24-00973) - [EMID:24613f2a05f75240]

On behalf of the authors, i would like to sincerely thank the reviewers for their great insight on this manuscript titled, *"Using Community-Based Participatory Approaches to Improve Access to Mass Drug Administration for Trachoma Elimination in a Pastoral Conflict Area of Kenya"*. Their comments and suggestions have greatly improved the quality of this paper.

In the revised manuscript, we have addressed all the comments and recommendations as pointed out. All changes are highlighted in the manuscript (highlighted version) and we have also attached a clean version of the document. In addition, we have provided point-by-point responses to the comments (see below).

Yours Sincerely,

Paul M Gichuki
Lead author

Reviewer 1

There are just a couple of main points that need addressing before finalizing for publication.

1. Description of Stage 2 in the methods:
    a. This section is critical to this paper as it focuses on methods related to the CBPR approach taken. Please provide some more details on how this was done. For example: what was the length of the workshop? Was the community involved in designing the workshop and if so how? What was done to develop relationships and build trust between the community, implementers and researchers (important to recognize at least these 3 main groups of stakeholders)? What were the power dynamics and how were these addressed? Were there any questions guiding the group work discussions? How were the report cards used? How were the challenges and solutions captured during the discussion (e.g. could all participants see what was being decided by putting results up on a board?).

Response: Thank you for this great insight, we have revised this section and addressed all the above issues in the same section (Lines 211-242)

    b. In my experience workshops like this can produce a much longer list of recommendations than are feasible to take action on - how were decisions made on which suggestions to prioritize?

Response: We have addressed this in Lines 238-243

    c. In addition to describing how the community was engaged in informing the design of the intervention the methods section I was looking for a description of how the community were engaged in the delivery of the intervention. I see this is in the results section and agree that it fits better in terms of flow where you have it. Maybe you could add a short note in the methods section to say this is described later.

Response: We agree with your suggestion and have addressed it in lines 241-243

2. Coverage estimates:
    a. Methods on how coverage was estimated before and after needs to be included in the methods section. If administrative coverage was used please note if the system has ever been evaluated e.g. using coverage surveys and or data quality assessments.

Response: We have addressed this in the methods section, lines 245-249

    b. In the results section, if this is based on surveys please include CIs and / or a statistical test of differences comparing years (this obviously does not apply if it is administrative coverage).

Response: Thank you, administrative coverage was used.

3. Considering limitations of the before and after (with no comparison group) design:
    a. It is important to add to the limitations section that this was a before and after design with no other comparison group. Given this design one cannot conclude with full certainty that the increase in coverage was due to changes made and not due to other changes in the environment or context.

Response: Thank you, we have addressed this under the study limitations section, Lines 459-467

    b. Given this limitation, care needs to be taken in the conclusions (and abstract) to not overly attribute the change in coverage to the intervention.

Response: Thank you, we have revised our conclusion (Lines 471-479) and abstract sections (Lines 42-44).

Costs: Given the big changes made to implementation, I am curious to know what the added cost of implementation was (if any)? If no costing data is available, it would be good to know if there were changes to budget lines and at the least add information on who funded the intervention?

Response: Thank you, we have included a section in the results section on cost implication (Lines 358-363).

Additionally, there are a few minor points to address:

Background:

- Line 68-9: more accurate to say blindness can be irreversible

Response: Thank you, this has been revised Line 69

- End line 79: minor editing of the sentence starting here is needed.

Response: Thank you, this has been revised Line 80

- Please note how geographic areas listed in paragraph starting line 105 relate to those listed starting on line 88

Response: Thank you, this has been revised Line 115

- Sentence starting line 20 seems to belong in the previous paragraph and is possibly repetition and can be deleted

Response: Thank you, this has been revised Line 69

- The paragraph goes on to say "Therefore, different strategies have been employed with an aim of engaging communities.."This would benefit by adding 1-2 examples.

Response: Thank you, this has been revised Line 129-134

- Paragraph starting line 26 - consider linking this more directly to community engagement. CBPR is not so much another approach, but one specific approach to foster community engagement …

Response: Thank you, this has been revised Line 132-146

- Please provide a short description of 'cattle rustling'.

Response: Thank you, we have added a brief description Line 110-11

Methods:

- Line 146: missing word

Response: Thank you, this has been revised Line 168

- Lines 145-151 are mostly repeated in the following paragraph

Response: Thank you, this section has been revised and the repeated section deleted (Lines 164-170)

- Line 163- sentence starting here needs revising.

Response: Thank you, this has been revised Line 177

- In table 1, first column- some entries are written as people and other are organizations e.g. I think CBO needs to be changed to CBO member. I also suggest sorting this table by the level at which the person operates (ward, county, etc)

Response: Thank you, we have revised the table (Line 207)

- Line 195: Section with heading "Stage 2…"
    - Consider revising the heading - I understanding that you are discussion how the initial findings were collected (and the response to the findings comes later)

Response: Thank you, we have revised the heading (Line 212)

Results:

- Lines 212-218: can save space here by referring to the table for summary and below for description on each of the key issues identified.

Response: Thank you, we have revised this section Lines 253-254

- Sentence 220- 223 needs editing.

Response: Thank you, the sentence has been edited Line 256-259

- Table 2 is very important, and it provides an excellent summary of the key points

Response: Thank you for the positive feedback

- Line 262: 'key strategies and interventions designed' Please clarify in this title and at the start of the section if these were only designed or also implemented? If there is a gap between designed and implemented what drove the decision making on what was implemented? Which items were implemented?

Response: Thank you, we have revised this section and addressed the concerns Line 298-303

- In this section I generally thought the table brought out the specificity of the adaptations made better than the text. Can consider editing the text to tighten it up, or referring more to the table and saving on the word count.

Response: Thank you, the sentence has been edited Line 256-259

- Section starting line 279 - please clarify in the description what was done differently to before? Were key messages changed to focus on specific messages? Was the community engaged differently in the delivery of the messages? Other studies have noted the importance of trusted messengers in communication (I see this discussed in the social mobilization section that follows). Which leads me to a related point that there seems to be a lot of overlap between community awareness and social mobilization

Response: Thank you, we have revised this section and addressed the overlapping areas 315-319

- Line 331- I am not sure I understand the first sentence

Response: Thank you, we have revised this sentence Line 364

Discussion:

- This discussion reiterates a lot of the findings and could possibly be edited for length.

Response: We have revised our discussion and reorganized it into focused sub sections

- Re sentence starting line 354. To reach a wider audience you might also consider referencing literature on 'co-creation' of interventions (and using that term in your key words) and linking this to 1 or 2 examples that go beyond NTDs.

Response: Thank you for this great insight, we have revised and added the idea of co-creation both in background (Lines 141-144) and discussion Line 378-381

References:

- Words are missing in line 556

This has been revised

Finally, I recently found this 2023 review by Riccardi et al on CBPR used to engage communities helpful and passing it along as I think it will be of interest to the authors: https://www.sciencedirect.com/science/article/abs/pii/S0168851023001902?via%3Dihub

Response: Thank you for referring this great review to us. We really appreciate.

Reviewer 2

The study addresses an important public health issue, but there are several areas that could benefit from revisions to improve clarity, coherence, and scientific rigor. Below are my detailed comments and recommendations:

1. Abstract:

1.1. Clarity and Focus (Line 1): The first sentence, "*Trachoma, a neglected tropical disease (NTD) is the leading infectious cause of blindness in sub-Saharan Africa*," is quite general and familiar to the journal's readership. I suggest rephrasing it to focus more on the study's context. For instance: "*In Baringo County, Kenya, trachoma remains endemic despite repeated mass drug administration (MDA) efforts, with coverage consistently falling short of WHO targets*."

Response: Thank you for the insight, we agree with your suggestion and have revised this part (Line 22-24)

1.2. Methods Section: The methods section of the abstract could be more explicit. Please specify the participatory approaches employed—was it qualitative? Were focus groups or surveys utilized? Briefly outline how stakeholders were identified and involved.

Response: We have revised the section and captured the missing information (Line 31-32)

1.3. Results Section: While the results are well-stated, the phrase *"community-based participatory approaches significantly increased trachoma MDA coverage"* is somewhat vague. If space allows, provide additional details on the specific interventions or changes that contributed to this improvement.

Response: We have revised this section (Line 42-44), however due to the limited space and the word limitation for this section, we have not been able to include all the information.

1.4. Conclusion: The conclusion is clear but would be strengthened by a sentence discussing the broader implications for future MDA campaigns or for other neglected tropical diseases (NTDs).

Response: Thank you, we have revised the conclusion to capture the raised concern (Line 43-44)

2. Introduction:

2.1. Clarifying the Knowledge Gap: While the introduction provides a broad overview of trachoma and its control, it would benefit from clearly articulating the knowledge gap this study addresses. What makes the participatory approach novel in this context compared to traditional MDA campaigns? Explicitly stating this would strengthen the study's rationale.

Response: thank you for this insight, we have addressed this in the background section, Lines 147-155

2.2. Emphasizing Participatory Approaches: The introduction could introduce community-based participatory research (CBPR) earlier and explain why it is particularly suited for this context. Additionally, highlighting how CBPR improves upon previous MDA strategies would better justify the need for this study.

Response: thank you for this insight, we have addressed this in the background section, Lines 129-146

2.3. Expand on Pastoralist Dynamics: The section on pastoralist migration patterns is essential for understanding the challenges of MDA delivery. Consider expanding this by discussing how migration affects public health interventions, potentially referencing similar studies on health delivery in migratory communities.

Response: We have expanded the section and addressed in background section, Lines 98-105

3. Discussion:

3.1. Improve Flow and Coherence: The discussion moves between various topics (stakeholder engagement, social mobilization, migration) without a clear structure. I recommend organizing the section into distinct sub-sections, such as "*Stakeholder Engagement*," "*Social Mobilization*," "*Logistical Planning*," and "*Addressing Pastoral Migration*."

Response: Thank you, we have re organized our discussion in to distinct sub sections, from Line 390

3.2. Repetition of Concepts: Some points, especially those related to social mobilization and communication strategies, are repeated. Consolidating these discussions will make the manuscript more concise and focused.

Response: We have consolidated the discussion points according to the sub themes and addressed the repetition.

3.3. Balancing Strengths and Challenges: While the discussion highlights the successes of the intervention, a more balanced perspective that acknowledges challenges and unexpected difficulties would strengthen the scientific rigor of the paper.

Response: Thank you, we have reviewed and also addressed some of the challenges faced throughout the discussion section.

3.4. Expand on the Role of Security and Migration: The involvement of security forces and the timing of the MDA campaign during the rainy season are presented as key strategies, but further explanation is needed. Did security involvement build community trust? Were there any resistance or challenges related to security? How did participation in the rainy season compare to past campaigns?

Response: We have addressed this under the sub section logistical planning Line 449-451**4.**

Limitations:

4.1. Broaden the Scope of Limitations: The limitation mentioned is valid but could be expanded to address additional points:

Response: We have expanded the scope of the study limitations and addressed this Lines 457-465

4.2. Selection Bias in Stakeholder Engagement: Were all community sub-groups adequately represented in the participatory process? If not, how might this have affected the outcomes?

Response: We have expanded the scope of the study limitations and addressed this Lines 457-465

4.3. Generalizability: Given that this study focuses on a specific pastoralist community, discuss how transferable the findings are to other regions or contexts, particularly those with different migration or conflict dynamics.

Response: Thank you, we have addressed this in our conclusion Line 471-474

4.4. Unintended Consequences: Were there any unintended negative outcomes from involving security forces, or other elements of the strategy? For instance, did the presence of security create tension or fear within certain population groups?

Response: There were no unintended negative outcomes from involving security teams, instead it served to instill confidence in the process (discussion Lines 449-451)

4.5. Long-Term Sustainability: Address the sustainability of these participatory approaches. Will the community maintain high levels of participation in future MDAs without continued intensive engagement?

Response: We have included this in the study limitation section 463-465

5. Conclusion: The last sentence, *"Community-based participatory approaches should be adopted for other health interventions,"* is somewhat broad. I suggest rephrasing it to be more specific: "*Future public health interventions, particularly those targeting neglected tropical diseases and community-driven health issues, should integrate participatory approaches to ensure long-term success and sustainability."*

Response: Thank you even for the suggestion which we have greatly adapted (Lines 446-478)
