# Supplementary material for: Using community-based participatory approaches to improve access to mass drug administration for trachoma elimination in a pastoral conflict area of Kenya
Source: PLoS Negl Trop Dis. 2024 Nov 11;18(11):e0012653. doi: 10.1371/journal.pntd.0012653 (PMC11581410; doi:10.1371/journal.pntd.0012653)
Supplement: Attachment — Submitted filename: PLOS NTDs Sept 2024.docx [file pntd.0012653.s001.docx]

**PLOS NTDs Sept 2024**

**Using Community-Based Participatory Approaches to Improve Access to Mass Drug Administration for Trachoma Elimination in a Pastoral Conflict Area of Kenya**

This paper reports on a before and after study that used community based participatory approaches to adapt the design of a mass drug administration (MDA) to increase access for a pastoral community living in a conflict areas in Kenya. The study is well thought out, well executed and well written. There is currently a big gap in the literature in both 1. Using participatory approaches to adapt MDA interventions and 2. Studies that report results of adapting interventions to increase access for marginalized populations. This study addresses both of these gaps.

This study is innovative and more like it are needed as many NTD programs reach the final mile and need to work out how to increase access for populations that have historically been left behind. The methods presented in this paper will serve to inform other programs facing similar issues. This paper will be of interest not just to policy makers, practitioners and researchers working in the NTD space but also to those working on other diseases using interventions that are also delivered through campaign approaches in the community, including those working on increasing access to COVID-19 vaccines.

There are just a couple of main points that need addressing before finalizing for publication.

1. Description of Stage 2 in the methods:
   a. This section is critical to this paper as it focuses on methods related to the CBPR approach taken. Please provide some more details on how this was done. For example: what was the length of the workshop? Was the community involved in designing the workshop and if so how? What was done to develop relationships and build trust between the community, implementers and researchers (important to recognize at least these 3 main groups of stakeholders)? What were the power dynamics and how were these addressed? Were there any questions guiding the group work discussions? How were the report cards used? How were the challenges and solutions captured during the discussion (e.g. could all participants see what was being decided by putting results up on a board?).
   b. In my experience workshops like this can produce a much longer list of recommendations than are feasible to take action on - how were decisions made on which suggestions to prioritize?
   c. In addition to describing how the community was engaged in informing the design of the intervention the methods section I was looking for a description of how the community were engaged in the delivery of the intervention. I see this is in the results section and agree that it fits better in terms of flow where you have it. Maybe you could add a short note in the methods section to say this is described later.
2. Coverage estimates:
   a. Methods on how coverage was estimated before and after needs to be included in the methods section. If administrative coverage was used please note if the system has ever been evaluated e.g. using coverage surveys and or data quality assessments.
   b. In the results section, if this is based on surveys please include CIs and / or a statistical test of differences comparing years (this obviously does not apply if it is administrative coverage).
3. Considering limitations of the before and after (with no comparison group) design:
   a. It is important to add to the limitations section that this was a before and after design with no other comparison group. Given this design one cannot conclude with full certainty that the increase in coverage was due to changes made and not due to other changes in the environment or context.
   b. Given this limitation, care needs to be taken in the conclusions (and abstract) to not overly attribute the change in coverage to the intervention.
4. Costs: Given the big changes made to implementation, I am curious to know what the added cost of implementation was (if any)? If no costing data is available it would be good to know if there were changes to budget lines and at the least add information on who funded the intervention?

Additionally, there are a few minor points to address:

Background:

- Line 68-9: more accurate to say blindness can be irreversible
- End line 79: minor editing of the sentence starting here is needed.
- Please note how geographic areas listed in paragraph starting line 105 relate to those listed starting on line 88
- Sentence starting line 20 seems to belong in the previous paragraph and is possibly repetition and can be deleted
- The paragraph goes on to say “Therefore, different strategies have been employed with an aim of engaging communities..”This would benefit by adding 1-2 examples.
- Paragraph starting line 26 - consider linking this more directly to community engagement. CBPR is not so much another approach, but one specific approach to foster community engagement …
- Please provide a short description of ‘cattle rustling’.

Methods:

- Line 146: missing word
- Lines 145-151 are mostly repeated in the following paragraph
- Line 163- sentence starting here needs revising.
- In table 1, first column- some entries are written as people and other are organizations e.g. I think CBO needs to be changed to CBO member. I also suggest sorting this table by the level at which the person operates (ward, county, etc)
- Line 195: Section with heading “Stage 2…”
  - Consider revising the heading - I understanding that you are discussion how the initial findings were collected (and the response to the findings comes later)

Results:

- Lines 212-218: can save space here by referring to the table for summary and below for description on each of the key issues identified.
- Sentence 220- 223 needs editing.
- Table 2 is very important, and it provides an excellent summary of the key points
- Line 262: ‘key strategies and interventions designed’ Please clarify in this title and at the start of the section if these were only designed or also implemented? If there is a gap between designed and implemented what drove the decision making on what was implemented? Which items were implemented?
- In this section I generally thought the table brought out the specificity of the adaptations made better than the text. Can consider editing the text to tighten it up, or referring more to the table and saving on the word count.
- Section starting line 279 - please clarify in the description what was done differently to before? Were key messages changed to focus on specific messages? Was the community engaged differently in the delivery of the messages? Other studies have noted the importance of trusted messengers in communication (I see this discussed in the social mobilization section that follows). Which leads me to a related point that there seems to be a lot of overlap between community awareness and social mobilization
- Line 331- I am not sure I understand the first sentence

Discussion:

- This discussion reiterates a lot of the findings and could possibly be edited for length.
- Re sentence starting line 354. To reach a wider audience you might also consider referencing literature on ‘co-creation’ of interventions (and using that term in your key words) and linking this to 1 or 2 examples that go beyond NTDs.

References:

- Words are missing in line 556

Finally, I recently found this 2023 review by Riccardi et al on CBPR used to engage communities helpful and passing it along as I think it will be of interest to the authors: https://www.sciencedirect.com/science/article/abs/pii/S0168851023001902?via%3Dihub
